# Prognostic value of red blood cell distribution width in traumatic brain injury: A mediation and deep learning analysis

**DOI:** 10.1371/journal.pone.0339879

**Published:** 2026-01-02

**Authors:** Shuting Ding, Zhen Zhang, Qifu Bo, Chenyu Ma, Minghao Wu, Xue Di, Manli Zhao, Kai Luo, Jiani Pan, Xin Zhang, Bingqiang Zhang, Suzhen Wang, Yujia Kong

**Affiliations:** 1 School of Public Health, Shandong Second Medical University, Weifang, China; 2 Department of Oncology, Affiliated Hospital of Shandong Second Medical University, Weifang, China; 3 Department of Encephalopathy, Traditional Chinese Medicine Hospital Affiliated to Shandong Second Medical University, Weifang, China; 4 Key Laboratory of Cancer and Immune Cells of Qingdao, Qingdao, China; University of Montenegro-Faculty of Medicine, MONTENEGRO

## Abstract

This study aimed to investigate the associations between age, red blood cell distribution width (RDW), and short-term mortality in patients with traumatic brain injury (TBI), with a particular focus on the role of RDW in mediating the impact of age on mortality. We conducted a retrospective cohort analysis of 1,203 ICU-admitted TBI patients from the MIMIC-IV database (v3.1). Cox proportional hazards regression, restricted cubic splines (RCS), and mediation analysis were employed to evaluate the relationships between age, RDW, and mortality outcomes. Both advanced age (adjusted hazard ratio [HR] = 1.022 for 28-day mortality; HR_adj_ = 1.031 for in-hospital mortality) and RDW (HR_adj_ = 1.085 for 28-day mortality; HR_adj_ = 1.094 for in-hospital mortality) were found to predict mortality (all *P* < 0.05) independently. RDW demonstrated a dose–response relationship with mortality: the highest quartile (Q4) exhibited a 2.061-fold increased risk of 28-day mortality (*P* = 0.010) and a 2.086-fold increased risk of in-hospital mortality (*P* = 0.022) compared to the lowest quartile (Q1). RCS analysis revealed significant nonlinear associations between age and 28-day mortality (*P* < 0.05) and between RDW and in-hospital mortality (*P* < 0.05). The mediation analysis demonstrated that RDW played a partial mediating role in age-related mortality, accounting for 4.40% of the total effect on 28-day mortality and 4.62% on in-hospital mortality (both *P* < 0.05). Deep learning survival models (e.g., Deepsurv: C-index: 0.759; IBS: 0.113; AUC (95% CI): 0.824 (0.735–0.900)) that incorporate age, RDW, and other clinical variables achieved robust predictive performance. Age and RDW are independent predictors of short-term mortality in TBI. RDW partially mediates the effect of age on TBI prognosis and shows potential as a practical biomarker for clinical risk stratification.

## Introduction

Traumatic brain injury (TBI) constitutes an ongoing public health concern worldwide, contributing significantly to mortality and long-term disability, particularly among young adults and the elderly [1,2]. Globally, TBI incidence reached 20.8 million new cases in 2021, corresponding to age-standardized rates of 259 per 100,000 population [3]. TBI typically stems from external mechanical forces, which trigger complex secondary pathophysiological cascades, including intracranial hemorrhage, disruption of the blood–brain barrier, and systemic inflammatory responses [4–6]. Notably, the ongoing demographic shift toward an aging global population exacerbates TBI incidence and severity in older adults [7,8], who tend to exhibit poorer outcomes, likely due to age-related physiological decline, comorbidities, and heightened susceptibility to secondary injury mechanisms [9].

Various prognostic factors have been identified in TBI, including injury severity (e.g., Glasgow Coma Scale [GCS] scores), neuroimaging findings, systemic physiological parameters, and emerging biomarkers [10,11]. Among these, red cell distribution width (RDW), a routinely measured hematologic index reflecting erythrocyte size variability, has gained traction as a prognostic marker in acute and chronic conditions [12–14]. Several investigations have examined the association between RDW and TBI. Elevated RDW has been reported as an independent risk factor for mortality in TBI patients [15,16]. One study found that non-surviving TBI patients had higher RDW levels at admission than survivors [17], whereas another study reported no significant differences [18]. Taken together, these conflicting results suggest that the relationship between RDW and mortality in TBI requires further investigation.

Age is a well-established prognostic determinant in TBI, with mortality and functional decline escalating markedly in older patients [19,20]. Neuroimaging studies further reveal that aging worsens TBI-related brain atrophy, reduces cortical thickness, and increases microstructural damage [21]. Nevertheless, the interplay between age and RDW in determining TBI outcomes remains underexplored. Specifically, it is unclear whether RDW mediates age-related increases in TBI mortality, with few studies addressing this causal pathway.

Given the complex and multifactorial nature of TBI outcomes [22], traditional regression methods may be insufficient to fully capture the nonlinear and high-dimensional relationships underlying these associations. In this context, recent advances in machine learning and deep learning–based survival models have provided practical tools to address these limitations. These approaches have been shown to improve prognostic accuracy and identify complex feature interactions in clinical prediction studies [23–25]. Integrating such methods may help generate additional insights into TBI prognosis beyond those obtained from conventional statistical analyses.

To address these gaps, we leveraged the MIMIC-IV database to investigate the interrelationships among age, RDW, and in-hospital/28-day mortality in TBI patients. Using causal mediation analysis, we examined whether RDW significantly mediates the effect of age on clinical outcomes, thus providing novel insights into prognostic mechanisms and potential avenues for risk stratification.

## Materials and methods

### Data source

This study is a retrospective analysis of the open-access Medical Information Mart for Intensive Care-IV (MIMIC-IV) database, which includes anonymized clinical data from patients treated at the Beth Israel Deaconess Medical Center between 2008 and 2022. The database was developed with approval from the Institutional Review Boards of both the Beth Israel Deaconess Medical Center and the Massachusetts Institute of Technology. Access was granted to the first author (Shuting Ding) after successful completion of mandatory online training and acceptance of the data use agreement (Certification Number: 62840007). The data used in this study were obtained from the publicly available MIMIC-IV database, which contains de-identified health-related data for critical care patients. Data extraction for the present analysis occurred from May 17 to June 20, 2025. As the dataset contained no identifiable patient information, further Institutional Review Board oversight was not required, and informed consent was waived. The research process strictly adhered to the Declaration of Helsinki and was reported in accordance with the Strengthening the Reporting of Observational Studies in Epidemiology (STROBE) guidelines.

### Study population

**Fig 1:**
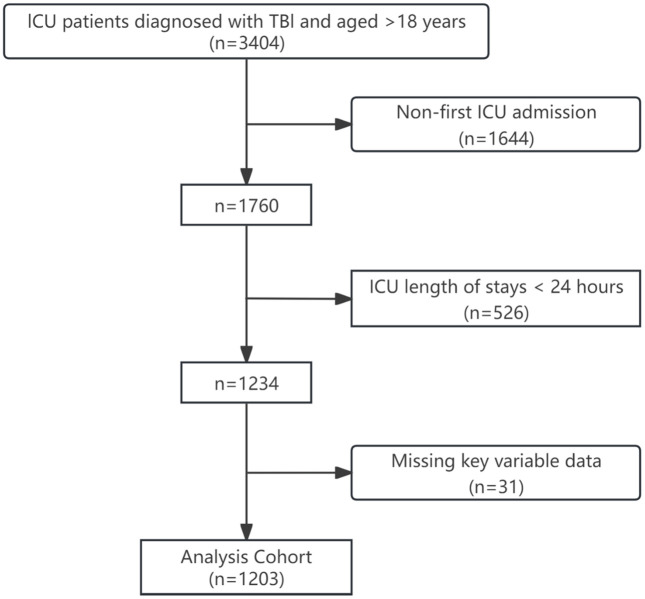
Flow diagram illustrating the selection of ICU patients diagnosed with TBI for inclusion in the analysis cohort. ICU: intensive care unit; TBI: Traumatic Brain Injury.

TBI patients were retrospectively identified from the MIMIC-IV database using ICD codes. The cohort included patients admitted to both hospital wards and the ICU. For individuals with multiple hospitalizations or ICU stays, only data from the first admission were analyzed. Participants were selected based on meeting all of the following criteria: (1) primary diagnosis of TBI at ICU admission, defied by ICD-9 codes (800–801, 803–804, 850–854, 950.1–950.3, 959.01) or ICD-10 codes (S02.0, S02.1, S02.8, S02.91, S04.02, S04.03, S04.04, S06, S07.1); (2) first ICU admission during the study period; and (3) age ≥ 18 years. Exclusion criteria: (1) missing critical data (e.g., RDW, mortality status); or (2) ICU length of stay < 24 hours (to avoid inclusion of rapidly discharged patients with incomplete data). The final cohort included 1203 patients with TBI, as shown in the patient selection process (Fig 1).

### Data extraction

Data were extracted using Structured Query Language (SQL) with PostgreSQL (version 14.8.1) and Navicat Premium (version 15). Variables included: Demographics (Gender, Race, Age, Weight,) Comorbidities (Cerebrovascular Disease, Dementia, Diabetes, Renal Disease), Clinical scores (Logistic Organ Dysfunction System [LODS], Sequential Organ Failure Assessment [SOFA], GCS), Laboratory parameters (RDW, White blood cell count [WBC], Sodium, Potassium, Chloride, Creatinine, Hemoglobin, Calcium, Blood urea nitrogen [BUN], Platelets, Hematocrit, Glucose), Interventions (Invasive mechanical ventilation [IMV], Furosemide, Cefazolin, Ceftriaxone, Vancomycin).

### Primary and secondary outcomes

Primary outcome: 28-day all-cause mortality. Secondary outcome: In-hospital all-cause mortality.

### Statistical analysis

Continuous variables were first assessed for normality using the Shapiro-Wilk test. Normally distributed variables were summarized as mean ± standard deviation (SD), while skewed variables were reported as median and interquartile range (IQR). Categorical variables were presented as counts and percentages. Comparisons between groups were performed using t-tests or ANOVA for normally distributed data, Mann-Whitney U or Kruskal-Wallis tests for non-normally distributed data, and Chi-square tests for categorical variables.

Cox proportional hazards regression modeled associations between RDW (categorized into quantiles Q1–Q4) and mortality: Model 1 included unadjusted estimates; Model 2 was adjusted for age; and Model 3 was adjusted for age, LODS, invasive ventilation, and WBC. Multicollinearity was evaluated using the variance inflation factor (VIF), with variables exceeding a VIF of 5 being excluded from the analysis. The remaining variables were retained for inclusion in the multivariable analysis (S1 Table). Restricted cubic spline (RCS) analysis with four knots positioned at the 5th, 35th, 65th, and 95th percentiles was applied to examine nonlinear associations of RDW and age with mortality, and to derive the corresponding thresholds for stratification.

Mediation analysis was conducted to assess the potential indirect effect of RDW on the relationship between age and 28-day mortality. We applied a causal steps framework, with age as the independent variable, RDW as the mediator, and mortality as the outcome. To obtain robust estimates of the indirect effect (a × b) and its uncertainty, we implemented non-parametric bootstrapping with 1,000 resamples. The 95% confidence interval for the indirect effect was calculated from the 2.5th and 97.5th percentiles of the bootstrap distribution. Mediation analysis were performed using Python (version 3.7) with the “statsmodels” and “scikit-learn” packages.

For missing data, variables with more than 15% missingness were excluded. Among the remaining variables, calcium (2.41%), admission weight (1.91%), and GCS (0.25%) had missing values. Multiple imputation by chained equations (MICE) was applied to variables with missingness rates of ≤ 15% using the “mice” package in R (version 4.4.1), with five imputations and 20 iterations. Logistic regression was applied to categorical data, and predictive mean matching to continuous data.

All statistical analyses were conducted using Python (version 3.7) and R (version 4.4.1). A two-tailed *P* < 0.05 was considered statistically significant.

### Model development and performance evaluation

To assess the predictive performance of the selected prognostic factors for TBI mortality, we employed Cox regression, random survival forest (RSF), and two deep learning–based survival models, Deepsurv and Coxtime. The dataset was randomly divided into training (70%), validation (15%), and testing (15%) sets. Model hyperparameters were optimized through five-fold cross-validation on the training data, and the corresponding search spaces are summarized in S2 Table. Model performance was evaluated using the time-dependent area under the ROC curve (AUC), concordance index (C-index), integrated Brier score (IBS), and calibration plots to comprehensively assess discrimination and calibration. All models were trained in Python (version 3.7). The Cox and RSF models were implemented using the “lifelines” and “scikit-survival” packages, respectively, while the deep learning models were constructed with “PyCox”.

## Results

### Population and baseline characteristics

A total of 3404 ICU patients diagnosed with TBI and aged > 18 years were initially screened. After restricting the cohort to patients admitted to the ICU for the first time (n = 1644), we further excluded those with ICU stays <24 hours (n = 526) and cases with missing key variable data (n = 31). Ultimately, 1,203 patients were included. Baseline characteristics stratified by 28-day and in-hospital mortality outcomes are summarized in Tables 1 and 2. The cohort had a median age of 68 years (IQR: 51–81.5), with 40.1% male (n = 483) and 59.9% female (n = 720), the majority (70.5%) identified as White.

**Table 1:** Baseline characteristics of participants stratified by 28-day survival.

Variable	Survivors (n = 1040)	Non-survivors (n = 163)	*P*
**Demographics**			
Gender			0.084
Male	407 (39.1%)	76 (46.6%)	
Female	633 (60.9%)	87 (53.4%)	
Race			0.057
White	740 (71.2%)	109 (66.9%)	
Black	63 (6.1%)	5 (3.1%)	
Other	237 (22.8%)	49 (30.1%)	
Age	67.0 (50.0–80.0)	79.0 (66.0–87.5)	**<0.001**
Weight	75.0 (63.5–87.1)	70.0 (58.0–80.0)	**<0.001**
**Comorbidities**			
CVD	132 (12.7%)	20 (12.3%)	0.981
Dementia	37 (3.6%)	8 (4.9%)	0.534
Diabetes	221 (21.2%)	31 (19.0%)	0.584
Renal disease	129 (12.4%)	23 (14.1%)	0.629
**Clinical scores**			
SOFA	3.0 (2.0–5.0)	5.0 (3.0–7.0)	**<0.001**
LODS	3.0 (2.0–5.0)	6.0 (4.0–7.0)	**<0.001**
GCS	14.0 (13.0–15.0)	15.0 (10.0–15.0)	0.933
**Laboratory parameters**			
RDW	13.9 (13.2–14.9)	14.9 (13.8–16.1)	<**0.001**
Sodium	138.0(136.0–140.0)	138.0 (135.0–140.0)	0.858
Potassium	3.8 (3.5–4.1)	3.7 (3.3–4.2)	0.332
Chloride	103.0 (100.0–106.0)	102.0 (99.0–107.0)	0.361
Creatinine	1.0 (0.8–1.2)	1.2 (0.9–1.6)	**<0.001**
Hemoglobin	11.0 (9.6–12.4)	10.1 (8.3–11.5)	**<0.001**
Calcium	8.3 (7.7–8.7)	8.2 (7.6–8.8)	0.741
BUN	17.0 (13.0–25.0)	23.0 (17.0–34.0)	**<0.001**
WBC	12.1 (8.7–16.0)	13.8 (9.6–18.9)	**0.001**
Platelets	179.0 (137.0–229.2)	162.0 (114.5–211.5)	**0.002**
Hematocrit	32.4 (28.1–36.4)	29.7 (24.4–33.8)	**<0.001**
Glucose	142.0 (117.0–173.0)	156.0 (129.0–194.0)	**<0.001**
**Interventions**			
IMV	472 (45.4%)	121 (74.2%)	**<0.001**
Furosemide	294 (28.3%)	39 (23.9%)	0.290
Cefazolin	259 (24.9%)	33 (20.2%)	0.233
Ceftriaxone	110 (10.6%)	18 (11.0%)	0.966
Vancomycin	264 (25.4%)	44 (27.0%)	0.733

CVD: Cardiovascular Disease; SOFA: Sequential Organ Failure Assessment; LODS: Logistic Organ Dysfunction Score; GCS: Glasgow Coma Scale; RDW: Red Cell Distribution Width; BUN: Blood Urea Nitrogen; WBC: White Blood Cell count; IMV: Invasive mechanical ventilation.

**Table 2:** Baseline characteristics of participants stratified by in-hospital survival.

Variable	Survivors (n = 1079)	Non-survivors (n = 124)	*P*
**Demographics**			
Gender			**0.023**
Male	421 (39.0%)	62 (50.0%)	
Female	658 (61.0%)	62 (50.0%)	
Race			**0.016**
White	769 (71.3%)	80 (64.5%)	
Black	65 (6.0%)	3 (2.4%)	
Other	245 (22.7%)	41 (33.1%)	
Age	67.0 (50.0–80.0)	78.0 (65.2–86.0)	**<0.001**
Weight	75.0 (63.0–87.0)	70.0 (59.5–80.6)	**0.003**
**Comorbidities**			
CVD	139 (12.9%)	13 (10.5%)	0.536
Dementia	39 (3.6%)	6 (4.8%)	0.667
Diabetes	229 (21.2%)	23 (18.5%)	0.564
Renal disease	135 (12.5%)	17 (13.7%)	0.812
**Clinical scores**			
SOFA	3.0 (2.0–5.0)	5.0 (3.0–7.0)	**<0.001**
LODS	3.0 (2.0–5.0)	6.0 (4.0–7.0)	**<0.001**
GCS	14.0 (13.0–15.0)	15.0 (9.8–15.0)	0.952
**Laboratory parameters**			
RDW	13.9 (13.2–15.0)	14.9 (13.8–16.4)	**<0.001**
Sodium	138.0 (136.0–140.0)	138.0 (135.0–140.0)	0.498
Potassium	3.8 (3.5–4.1)	3.7 (3.3–4.1)	0.106
Chloride	103.0 (100.0–106.0)	102.0 (99.0–107.0)	0.329
Creatinine	1.0 (0.8–1.2)	1.2 (0.8–1.6)	**<0.001**
Hemoglobin	11.0 (9.5–12.4)	10.1 (8.4–11.5)	**<0.001**
Calcium	8.3 (7.7–8.7)	8.2 (7.6–8.8)	0.900
BUN	17.0 (13.0–25.0)	24.0 (17.8–34.0)	**<0.001**
WBC	12.2 (8.7–16.2)	13.3 (9.5–18.2)	**0.029**
Platelets	179.0 (135.5–228.0)	164.0 (105.8–213.8)	**0.010**
Hematocrit	32.3 (27.9–36.2)	30.2 (25.1–34.6)	**<0.001**
Glucose	142.0 (117.0–173.0)	161.0 (129.0–198.8)	**<0.001**
**Interventions**			
IMV	493 (45.7%)	100 (80.6%)	**<0.001**
Furosemide	305 (28.3%)	28 (22.6%)	0.217
Cefazolin	271 (25.1%)	21 (16.9%)	0.057
Ceftriaxone	114 (10.6%)	14 (11.3%)	0.925
Vancomycin	275 (25.5%)	33 (26.6%)	0.870

CVD: Cardiovascular Disease; SOFA: Sequential Organ Failure Assessment; LODS: Logistic Organ Dysfunction Score; GCS: Glasgow Coma Scale; RDW: Red Cell Distribution Width; BUN: Blood Urea Nitrogen; WBC: White Blood Cell count; IMV: Invasive mechanical ventilation.

The trends observed for 28-day and in-hospital mortality outcomes were generally consistent. Non-survivors exhibited significantly different characteristics compared to survivors (all *P* < 0.05): older age, higher invasive ventilation use. Laboratory comparisons revealed significant intergroup differences in multiple biomarkers. Non-survivors exhibited elevated levels of RDW, creatinine, BUN, WBC, and glucose compared to survivors (all *P* < 0.05). Conversely, hemoglobin, hematocrit, and platelet counts were notably reduced in non-survivors (all *P* < 0.05). No statistically significant differences were observed for calcium, sodium, potassium, or chloride levels (*P* > 0.05).

### Cox regression analyses

Univariate and multivariate Cox regression analyses were conducted to identify prognostic factors for 28-day and in-hospital mortality. The multivariate model showed that age (HR: 1.022, 95% CI: 1.012–1.033; *P* < 0.001 for 28-day mortality; HR: 1.031, 95% CI: 1.018–1.045; *P* < 0.001 for in-hospital mortality) and RDW levels (HR: 1.085, 95% CI: 1.006–1.169; *P* = 0.034 for 28-day mortality; HR: 1.094, 95% CI: 1.008–1.188; *P* = 0.032 for in-hospital mortality) were significant independent predictors for mortality. Additionally, LODS (HR: 1.175, 95% CI: 1.074–1.285; *P* < 0.001 for 28-day mortality; HR: 1.165, 95% CI: 1.051–1.292; *P* = 0.004 for in-hospital mortality), WBC (HR: 1.015, 95% CI: 1.001–1.029; *P* = 0.033 for in-hospital mortality), and IMV (HR: 2.551, 95% CI: 1.725–3.772; *P* < 0.001 for 28-day mortality; HR: 2.762, 95% CI: 1.687–4.522; *P* < 0.001 for in-hospital mortality) were also demonstrated significantly associated with mortality risk (Table 3).

**Table 3:** Univariate and multivariate Cox regression analyses of factors associated with mortality.

Variables	Univariate Cox analysis		Multivariate Cox analysis	
	HR(95%CI)	*P*	HR(95%CI)	*P*
28-day mortality				
Age	1.022 (1.012–1.031)	**<0.001**	1.022 (1.012–1.033)	**<0.001**
Gender (M vs. F)	1.405 (1.032–1.648)	**0.031**	1.213 (0.850–1.729)	0.287
GCS	0.923 (0.881–0.968)	**0.001**	1.001 (0.950–1.055)	0.966
SOFA	1.160 (1.104–1.219)	**<0.001**	0.996 (0.924–1.075)	0.927
LODS	1.237 (1.178–1.298)	**<0.001**	1.175 (1.074–1.285)	**<0.001**
BUN	1.011 (1.005–1.017)	**0.001**	0.999 (0.989–1.008)	0.770
Weight	0.988 (0.979–0.996)	**0.005**	0.992 (0.982–1.002)	0.110
RDW	1.159 (1.082–1.241)	**<0.001**	1.085 (1.006–1.169)	**0.034**
IMV	2.596 (1.826–3.691)	**<0.001**	2.551 (1.725–3.772)	**<0.001**
WBC	1.019 (1.008–1.031)	**0.001**	1.015 (1.001–1.029)	**0.033**
In-hospital mortality				
Age	1.035 (1.023–1.047)	**<0.001**	1.031 (1.018–1.045)	**<0.001**
Gender (M vs. F)	1.789 (1.253–2.553)	**0.001**	1.428 (0.953–2.141)	0.084
GCS	0.929 (0.882–0.979)	**0.006**	0.996 (0.942–1.054)	0.881
SOFA	1.152 (1.092–1.216)	**<0.001**	0.995 (0.913–1.084)	0.913
LODS	1.245 (1.177–1.315)	**<0.001**	1.165 (1.051–1.292)	**0.004**
BUN	1.016 (1.009–1.023)	**<0.001**	1.003 (0.992–1.014)	0.643
Weight	0.984 (0.975–0.994)	**0.002**	0.993 (0.982–1.005)	0.246
RDW	1.185 (1.100–1.284)	**<0.001**	1.094 (1.008–1.188)	**0.032**
IMV	2.543 (1.614–4.005)	**<0.001**	2.762 (1.687–4.522)	**<0.001**

M: male; F: female; SOFA: Sequential Organ Failure Assessment; LODS: Logistic Organ Dysfunction Score; GCS: Glasgow Coma Scale; RDW: Red Cell Distribution Width; BUN: Blood Urea Nitrogen; IMV: Invasive mechanical ventilation; WBC: White Blood Cell count.

### Kaplan-meier survival analysis

**Fig 2:**
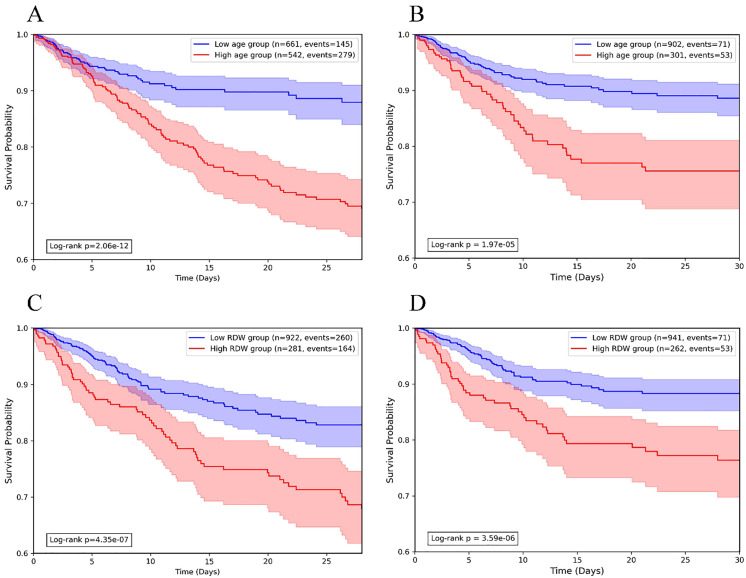
Kaplan–Meier survival curves for age and RDW levels to 28-day and in-hospital mortality among patients with TBI. (A) Age and 28-day mortality; (B) Age and in-hospital mortality; (C) RDW levels and 28-day mortality; (D) RDW levels and in-hospital mortality. TBI, traumatic brain injury; RDW, red cell distribution width.

Kaplan-Meier (KM) survival analysis was employed to evaluate mortality in TBI patients of different ages and RDW levels. Age stratification analysis revealed that the 28-day mortality rate was significantly higher in the older age group (n = 542, events = 279) compared to the younger age group (n = 663, events = 145; *P* = 2.06 × 10 ⁻ ¹²; Fig 2A). Similarly, for in-hospital mortality, the older group (n = 381, events = 53) demonstrated a significantly higher rate relative to the younger group (n = 903, events = 71, *P* = 1.97 × 10 ⁻ ⁵; Fig 2B).

RDW stratification analysis showed that, for 28-day mortality analysis, 15.30% was determined as the optimal cutoff value for RDW. The high-RDW group (n = 281, events = 164) exhibited a higher mortality than the low-RDW group (n = 923, events = 260, *P* = 4.35 × 10 ⁻ ⁷; Fig 2C). Regarding the in-hospital mortality analysis, a cutoff value of 15.40% was defined as the optimal RDW value. The high-RDW group (n = 262, events = 53) had a significantly higher mortality compared to the low-RDW group (n = 941, events = 71, *P* = 3.59× 10 ⁻ ⁶; Fig 2D).

### Cox regression analysis of RDW and TBI mortality

As presented in Table 4, RDW levels demonstrated significant associations with both 28-day and in-hospital mortality. Three hierarchical models were constructed. In the fully adjusted Model 3, RDW was independently associated with a higher risk of 28-day mortality (HR = 1.088, 95% CI: 1.011–1.170, *P* = 0.020) and in-hospital mortality (HR = 1.102, 95% CI: 1.017–1.195, *P* = 0.017). To evaluate the dose–response relationship, RDW levels were stratified into quartiles (Q1–Q4), with Q1 serving as the reference group. In the unadjusted model, patients in the highest quartile (Q4) had significantly increased risks of both 28-day mortality (HR = 3.453, 95% CI: 2.030–5.873, *P* < 0.001) and in-hospital mortality (HR = 3.910, 95% CI: 2.133–7.169, *P* < 0.001). In the fully adjusted model (Model 3), Q4 was associated with higher risks of both 28-day mortality (HR = 2.061, 95% CI: 1.192–3.562, *P* = 0.008) and in-hospital mortality (HR = 2.086, 95% CI: 1.114–3.906, *P* = 0.028). Statistically significant linear trends across quartiles were observed (*P*_trend_ = 0.014 for 28-day mortality; *P*_trend_ = 0.033 for in-hospital mortality).

**Table 4:** The association between RDW levels and mortality analyzed using Cox analyses.

	Model 1		Model 2		Model 3	
	HR (95% CI)	P-value	HR (95% CI)	P-value	HR (95% CI)	P-value
28-day mortality						
RDW	1.159 (1.082, 1.241)	**<0.001**	1.144 (1.066, 1.228)	**<0.001**	1.088 (1.011, 1.170)	**0.020**
RDW(Quartile) (Q1 as reference)						
Q2	2.202 (1.236, 3.921)	**0.007**	1.997 (1.119, 3.562)	**0.019**	1.658 (0.924, 2.973)	**0.077**
Q3	2.007 (1.135, 3.550)	**0.017**	1.817 (1.025, 3.219)	**0.041**	1.444 (0.809, 2.578)	0.183
Q4	3.453 (2.030, 5.873)	**<0.001**	2.903 (1.698, 4.963)	**<0.001**	2.061 (1.192, 3.562)	**0.008**
*P* for trend		**<0.001**		**<0.001**		**0.014**
In-hospital mortality						
RDW	1.185 (1.100, 1.277)	**<0.001**	1.161 (1.073, 1.255)	**<0.001**	1.102 (1.017, 1.195)	**0.017**
RDW(Quartile) (Q1 as reference)						
Q2	2.287 (1.175, 4.451)	**0.015**	1.966 (1.008, 3.834)	**0.047**	1.687 (0.859, 3.315)	0.176
Q3	2.234 (1.168, 4.272)	**0.015**	1.895 (0.989, 3.630)	0.054	1.607 (0.832, 3.102)	0.192
Q4	3.910 (2.133, 7.169)	**<0.001**	2.940 (1.595, 5.419)	**0.001**	2.086 (1.114, 3.906)	**0.028**
*P* for trend		**<0.001**		**0.001**		**0.033**

Model 1: unadjusted; Model 2: adjusted for age; Model 3: adjusted for age, LODS, IMV, WBC.

HR: hazard ratio; CI: confidence interval; RDW: red cell distribution width; LODS: Logistic Organ Dysfunction Score; IMV: Invasive mechanical ventilation; WBC: white blood cell count.

### Non-linear analyses

**Fig 3:**
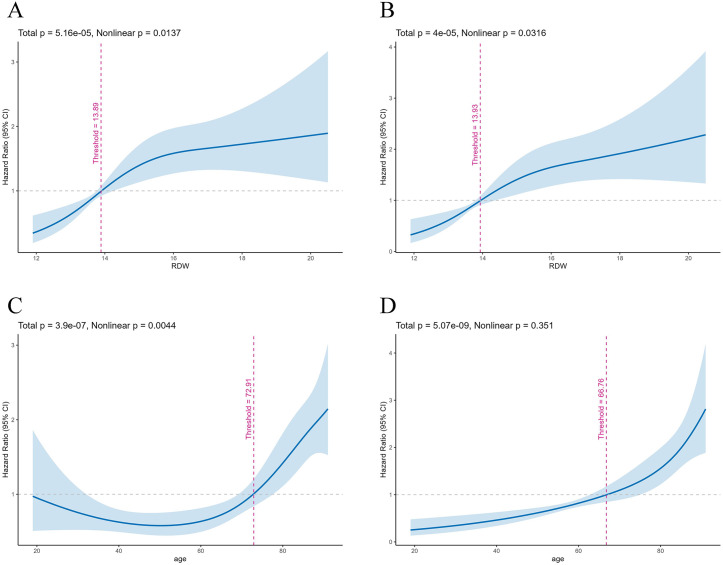
RCS analysis of RDW and age in relation to 28-day and in-hospital mortality among patients with TBI. (A) Association between RDW and 28-day mortality; (B) Association between RDW and in-hospital mortality; (C) Association between age and 28-day mortality; (D) Association between age and in-hospital mortality. The pink dashed line indicates the threshold identified by the RCS analysis.

RCS analysis revealed a significant nonlinear association between RDW levels and in-hospital mortality (*P*_nonlinearity_ < 0.05; Fig 3A). The risk of mortality exhibited a progressive increase with rising RDW values, with a pronounced acceleration beyond the reflection point; the risk increased significantly faster, with a nonlinear trend that was statistically significant (*P*_nonlinearity_ < 0.05). In contrast, although RDW levels demonstrated a positive trend over 28 days, no considerable nonlinearity was observed (Fig 3B).

Further analysis of the age-related mortality revealed a robust nonlinear relationship between age and 28-day mortality (*P*_nonlinearity_* *< 0.05; Fig 3C). The curve indicated a steep escalation in mortality risk beyond 72.91 years, indicating a threshold effect of age on short-term prognosis. Regarding in-hospital mortality, the age-related curve displayed a gradual upward slope. Although the overall risk increased with age, the nonlinear trend was relatively feeble (*P*_nonlinearity_ > 0.05; Fig 3D).

### Mediation role of RDW in the association between age and mortality among TBI patients

**Fig 4:**
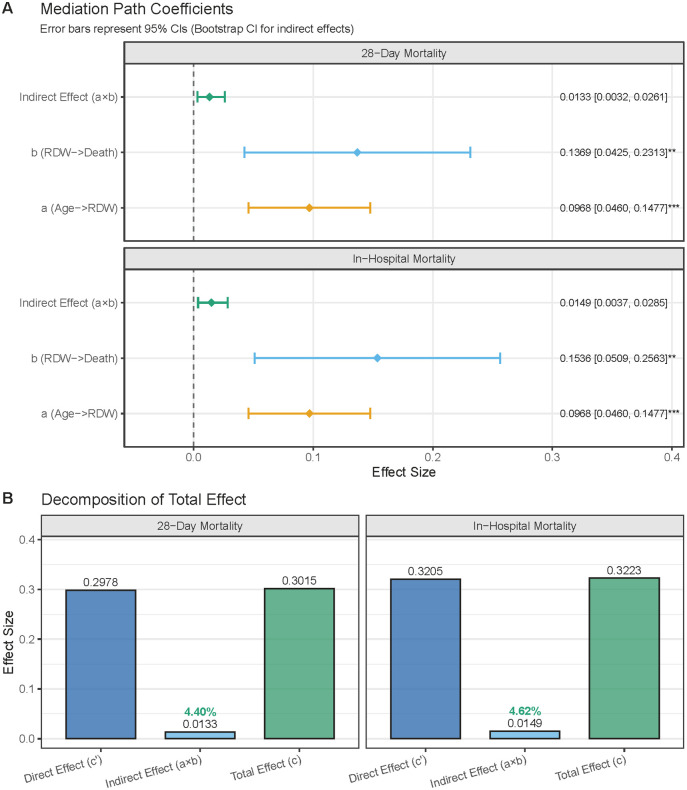
Mediation Analysis of Age, Red Cell Distribution Width (RDW), and Mortality in TBI Patients. (A) Mediation Path Coefficients. Indirect Effect (a × b): The combined effect of age on mortality mediated through RDW; b (RDW→Death): Direct effect of RDW on mortality; a (Age → RDW): Effect of age on RDW. (B) Decomposition of Total Effect. Direct Effect (c’): The direct effect of age on mortality, accounting for the mediating role of RDW; Indirect Effect (a × b): The proportion of the total effect mediated by RDW; Total Effect (c): The overall effect of age on mortality.

Our analysis revealed a significant correlation among age, RDW levels, and both 28-day and in-hospital mortality in TBI patients. To enhance clinical interpretability, age was standardized as a continuous variable scaled in 10-year increments. Mediation analysis was employed to evaluate the potential mediating effect of RDW on the association between age (increased by 10 years) and TBI mortality. For 28-day mortality, RDW partially mediated the effect of age on mortality. The average causal mediation effect (ACME) reached statistical significance (95% bootstrap CI: 0.0032–0.0261), indicating a meaningful indirect pathway via RDW. The average direct effect (ADE) of age remained significant (estimate: 0.2978; 95% CI: 0.1831–0.4125; *P* = 3.597 × 10^−7^), as did the total effect (estimate: 0.3015; 95% CI: 0.1881–0.4149; *P* = 1.878 × 10^−7^). RDW accounted for 4.40% of the total effect size (Fig 4A). For in-hospital mortality, similar mediation patterns were observed. The ACME was statistically significant (95% bootstrap CI: 0.0037–0.0285), indicating an indirect effect of age on mortality via RDW. The ADE (estimate: 0.3205; 95% CI: 0.1894–0.4516; *P* = 1.649 × 10^−6^) and the total effect (estimate: 0.3242; 95% CI: 0.1931–0.4516; *P* = 1.021 × 10^−6^) both remained significant. The proportion of the total effect mediated by RDW was 4.62% (Fig 4B).

### Prognostic modeling of TBI using traditional and deep learning approaches

**Fig 5:**
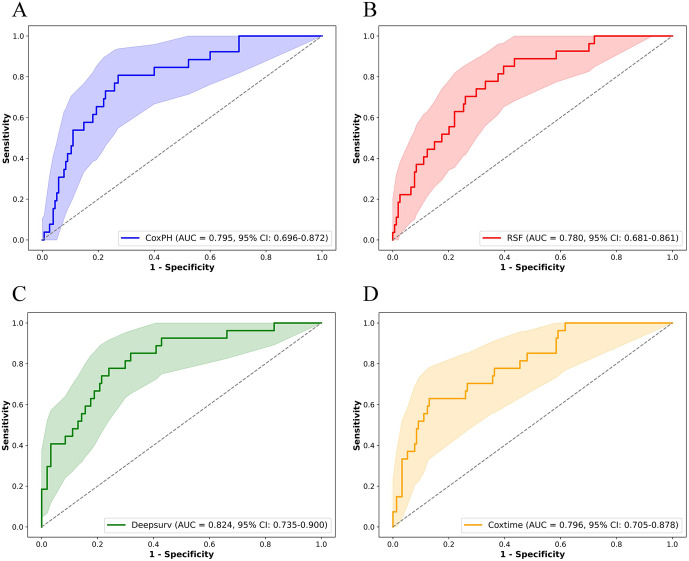
ROC curves for the four survival models. (A) CoxPH; (B) RSF; (C) Deepsurv; (D) Cox-time. The shaded area represents the 95% confidence interval of the AUC. AUC: area under the curve; CI: confidence interval.

**Fig 6:**
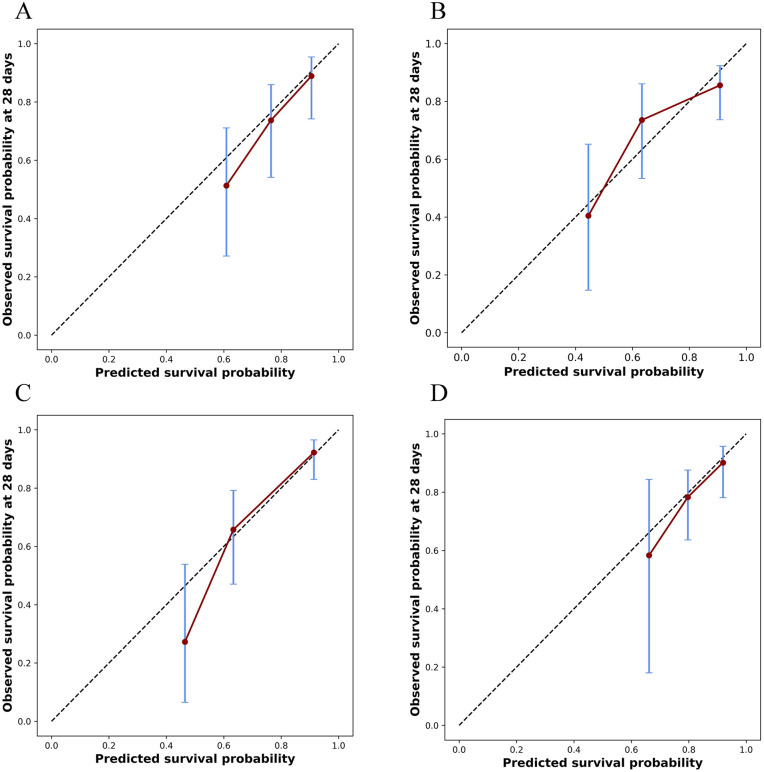
Calibration curves for predicting 28-day survival probability using four models. (A) CoxPH; (B) RSF; (C) Deepsurv; (D) Cox-time. The red solid line indicates the calibration performance of each model. The blue error bars represent the 95% confidence intervals of the observed survival probability for each prediction group.

The covariates identified through multivariable Cox regression analysis—including age, RDW, LODS, WBC, and IMV—demonstrated robust predictive performance across four survival models: Cox, RSF, DeepSurv, and Cox-Time. The DeepSurv model achieved the highest predictive accuracy (C-index: 0.759; IBS: 0.113; AUC (95% CI): 0.824 (0.735–0.900)), followed by the Cox-Time model (C-index: 0.716; IBS: 0.113; AUC (95% CI): 0.796 (0.705–0.878)), the Cox model (C-index: 0.743; IBS: 0.124; AUC (95% CI): 0.795 (0.696–0.872)), and the RSF model (C-index: 0.613; IBS: 0.122; AUC (95% CI): 0.780 (0.681–0.861)).The ROC curves (Figs 5A–D) confirmed the discriminative capacity of each model, and the calibration plots (Figs 6A–D) indicated good agreement between the predicted and observed probabilities of 28-day mortality.

## Discussion

TBI remains a substantial global public health challenge, contributing significantly to mortality and long-term disability [1,2]. In our study of ICU-admitted TBI patients, age and RDW were identified as independent predictors of short-term mortality. Notably, RDW demonstrated a significant dose–response association with mortality risk and partially mediated the impact of age on death. These findings underscore the prognostic importance of RDW in TBI and support its use as a practical biomarker for clinical risk stratification and management.

Consistent with previous observational and registry-based studies, our analysis reaffirms age as an independent and powerful predictor of adverse outcomes following TBI [19,20]. In both 28-day and in-hospital mortality models, older age was associated with a higher risk of death. This association remained significant after adjusting for injury severity, organ dysfunction scores, and physiological derangements. Importantly, our data further reveal that this relationship between age and 28-day mortality is nonlinear, with mortality risk escalating more sharply in advanced age groups. This age-related vulnerability is likely to be caused by several biological and clinical factors. This pattern may reflect age-related decline in neuroplasticity, reduced compensatory capacity, and heightened susceptibility to secondary insults such as inflammation and organ dysfunction [26–28]. Ageing is accompanied by a decline in neuroplasticity and regenerative capacity, an increased prevalence of comorbidities, polypharmacy, and diminished physiological reserve. These age-related changes, which are common in older adults, can compromise the response to acute brain injury [29,30]. Additionally, the ageing brain exhibits a distinct immune-inflammatory response characterized by chronic microglial activation and impaired resolution of inflammation, which can exacerbate secondary brain injury [31]. These considerations emphasize the importance of age-specific management strategies and identifying modifiable factors that can mitigate age-related risks.

RDW, a measure of the heterogeneity of circulating erythrocyte size, has emerged in recent years as a prognostic biomarker in diverse critical illnesses, ranging from cardiovascular disease to sepsis and ARDS [12–14,32]. Our analysis demonstrates that RDW is independently associated with both 28-day and in-hospital mortality in TBI patients. Observational studies have demonstrated that increased RDW is independently associated with adverse outcomes in ICU patients [33]. Moreover, a retrospective study found that in patients with head trauma–induced loss of consciousness, higher RDW was associated with an increased risk of brain death, suggesting it may serve as an early prognostic marker [34]. Biologically, elevated RDW appears to reflect interrelated pathophysiological processes, including systemic inflammation, oxidative stress, and impaired erythropoiesis, all of which contribute to the progression of secondary brain injury following TBI. Inflammatory cytokines can interfere with erythropoiesis, alter red blood cell membrane integrity, and promote anisocytosis, resulting in higher RDW values [35]. RDW may also serve as an indirect marker of underlying chronic disease burden or nutritional deficiencies, both of which themselves are associated with worse outcomes in critical illness [36]. Additionally, red blood cell deformability and rheology affect microcirculatory perfusion and oxygen delivery to injured brain tissue, suggesting that elevated RDW could contribute to tissue hypoxia and further neurological deterioration [15,37].

Our study also showed that RDW slightly mediates the effect of age on mortality in TBI patients. Patel et al. reported that RDW was strongly associated with mortality from multiple causes among middle-aged and older adults, each 1% increase in RDW was associated with approximately a 14% higher risk of death [38]. Additionally, studies in geriatric patients indicated that RDW is a dynamic variable, progressively increasing in the years preceding death, particularly during the last year of life, further supporting its role as a marker of physiological aging and mortality risk [39]. Moreover, hematologic studies have shown that RDW varies with age and reflects immunologic and inflammatory alterations linked to increased mortality risk [40]. These findings suggest that RDW may represent one hematologic pathway through which aging contributes to worse outcomes after TBI, reflecting a biologically plausible mechanism linking aging, hematologic dysregulation, and mortality risk.

Our analysis also reveals that RDW partially mediates impact of age on mortality risk in patients with TBI. Although the mediated proportion is modest, it was statistically significant and biologically meaningful, suggesting that elevated RDW may represent one path through which aging confers increased vulnerability after TBI. This finding is supported by prior studies. One study suggests that the impact of aging on inflammation and oxidative stress may influence patient prognosis by altering erythrocyte morphology, including RDW [38]. Another investigation highlights RDW as a hematologic marker that reflects chronic inflammatory status, considered a crucial mechanism underlying the risk of age-related diseases [41]. Recent evidence suggests that age-related alterations in blood parameters, including RDW, are associated with increased mortality risk. These findings highlight the potential of RDW as a biomarker reflecting systemic aging processes and their detrimental health effects [42].

In recent years, deep learning-based survival models have become increasingly valuable for predicting clinical outcomes, particularly in complex and heterogeneous conditions [43,44]. We applied four survival models to assess the prognostic value of the significant variables identified in our analysis. Deep learning–based survival analysis models, designed to handle time-to-event data and nonlinear relationships, demonstrated strong discriminative performance in predicting 28-day mortality. Notably, RDW emerged as a consistent and robust predictor across all models, reinforcing its potential as a clinically valuable biomarker for risk stratification in TBI patients. Existing prognostic models, such as the IMPACT and CRASH models [45,46], are widely used for TBI outcome prediction. As a readily available and inexpensive hematological parameter, RDW may provide complementary information to existing models, future studies could explore the incremental predictive value of incorporating RDW into the IMPACT and CRASH models to evaluate its potential role in risk stratification and clinical decision-making. Moreover, it may be valuable to assess the incremental prognostic contribution of RDW in combination with widely used TBI indicators, such as GCS, pupillary response, and CT-based scoring systems.

These findings offer practical implications for improving prognostic assessment in TBI. RDW, as a widely available and inexpensive biomarker, could be readily incorporated into clinical risk models to enhance early identification of high-risk patients. The combined evaluation of age and RDW may further refine prognostic accuracy, particularly in older adults. The observed nonlinear association between RDW and mortality suggests that even modest elevations may be clinically meaningful, underscoring the need to establish appropriate thresholds. Finally, the application of deep learning survival models demonstrates the potential of integrating routine clinical variables into advanced predictive frameworks to support individualized TBI management.

However, several limitations should be acknowledged. First, its retrospective design may introduce selection bias and unmeasured confounding. Second, as the analysis was conducted using a single-center dataset, the generalizability of the results to other populations or settings may be limited. Third, although we adjusted for numerous confounders, residual confounding due to unmeasured factors cannot be excluded. Fourth, RDW was measured only at ICU admission, longitudinal changes in RDW and their association with outcomes were not assessed. Finally, while mediation analysis provides insight into potential causal pathways, it cannot establish causality due to the observational nature of the data.

Further research is warranted to validate these findings in larger, multi-center cohorts with diverse populations and to explore the potential mechanisms linking RDW, aging, and TBI outcomes. Prospective studies examining dynamic changes in RDW over time, as well as interventional trials targeting modifiable factors that contribute to elevated RDW, may provide insight into potential therapeutic avenues. In addition, future studies should collect more comprehensive data on factors such as nutritional status, chronic anemia, blood transfusions, injury severity, comorbidities, and interventions, in order to further clarify these associations.

## Conclusions

In summary, this study highlights the prognostic relevance of RDW in TBI patients, both as an independent predictor of short-term mortality and as a partial mediator of the age-mortality relationship. Our findings demonstrate that RDW, a routinely available hematologic parameter, provides meaningful prognostic information beyond established clinical indicators. The consistent performance of RDW across traditional and deep learning-based survival models underscores its potential utility in enhancing individualized risk assessment. Incorporating RDW into clinical decision-making frameworks may facilitate early identification of high-risk patients and inform age-sensitive management strategies. These results warrant further validation and mechanistic exploration to better define the role of RDW in the complex pathophysiology of TBI.

## Supporting information

S1 Table VIF of Candidate Variables for Multivariable Cox Regression. The table presents the Variance Inflation Factor (VIF) for candidate variables considered in the multivariable Cox regression analysis. Variables with VIF > 5 were considered highly collinear and excluded from the final model. VIF: variance inflation factor; SAPSII: Simplified Acute Physiology Score II; LODS: Logistic Organ Dysfunction System; SOFA: Sequential Organ Failure Assessment; BUN: Blood Urea Nitrogen; GCS: Glasgow Coma Scale; IMV: Invasive mechanical ventilation; RDW: red blood cell distribution width; WBC: white blood cell count.
(DOCX)

S2 Table The hyper-parameters search space of ML model. The table summarizes the hyper-parameter search ranges used for three machine learning survival models: Random Survival Forest (RSF), DeepSurv, and Cox-Time. For each model, the hyper-parameters and their corresponding candidate values used in the grid search or optimization procedure are listed. ML: Machine Learning; RSF: Random Survival Forest.
(DOCX)
