# Supplementary material for: Prognostic value of red blood cell distribution width in traumatic brain injury: A mediation and deep learning analysis
Source: PLoS One. 2026 Jan 2;21(1):e0339879. doi: 10.1371/journal.pone.0339879 (PMC12758782; doi:10.1371/journal.pone.0339879)
Supplement: S1 Table — The table presents the Variance Inflation Factor (VIF) for candidate variables considered in the multivariable Cox regression analysis. Variables with VIF > 5 were considered highly collinear and excluded from the final model. VIF: variance inflation factor; SAPSII: Simplified Acute Physiology Score II; LODS: Logistic Organ Dysfunction System; SOFA: Sequential Organ Failure Assessment; BUN: Blood Urea Nitrogen; GCS: Glasgow Coma Scale; IMV: Invasive mechanical ventilation; RDW: red blood cell distribution width; WBC: white blood cell count. (DOCX) [file pone.0339879.s001.docx]

| **Table S1.** VIF of Candidate Variables for Multivariable Cox Regression. | | |
| --- | --- | --- |
| **Variable** | **VIF** | **Note** |
| Hemoglobin | 14.28 | VIF > 5, excluded |
| Hematocrit | 13.14 | VIF > 5, excluded |
| SAPSII | 5.11 | VIF > 5, excluded |
| LODS | 4.14 | Retained |
| Age | 2.40 | Retained |
| SOFA | 2.28 | Retained |
| BUN | 1.63 | Retained |
| GCS | 1.60 | Retained |
| IMV | 1.48 | Retained |
| RDW | 1.42 | Retained |
| Gender | 1.22 | Retained |
| Weight | 1.12 | Retained |
| WBC | 1.12 | Retained |
| The table presents the Variance Inflation Factor (VIF) for candidate variables considered in the multivariable Cox regression analysis. Variables with VIF > 5 were considered highly collinear and excluded from the final model. VIF: variance inflation factor; SAPSII: Simplified Acute Physiology Score II; LODS: Logistic Organ Dysfunction System; SOFA: Sequential Organ Failure Assessment; BUN: Blood Urea Nitrogen; GCS: Glasgow Coma Scale; IMV: Invasive mechanical ventilation; RDW: red blood cell distribution width; WBC: white blood cell count | | |
