# Supplementary material for: Prognostic value of red blood cell distribution width in traumatic brain injury: A mediation and deep learning analysis
Source: PLoS One. 2026 Jan 2;21(1):e0339879. doi: 10.1371/journal.pone.0339879 (PMC12758782; doi:10.1371/journal.pone.0339879)
Supplement: S2 Table — The table summarizes the hyper-parameter search ranges used for three machine learning survival models: Random Survival Forest (RSF), DeepSurv, and Cox-Time. For each model, the hyper-parameters and their corresponding candidate values used in the grid search or optimization procedure are listed. ML: Machine Learning; RSF: Random Survival Forest. (DOCX) [file pone.0339879.s002.docx]

| **Table S2.** The hyper-parameters search space of ML models. | | |
| --- | --- | --- |
| **Model hyper-parameter** | | **Hyper-parameter search space** |
| **RSF** | |  |
|  | n_estimator | [50,100,200], |
|  | max_depth | [10,20,50] |
|  | min_sample_split | [1,3,5,7,10] |
|  | min_sample_leaf | [1,3,5,7,10] |
| **DeepSurv** | |  |
|  | num_node | [64, 32], [64, 64],[64,128] |
|  | dropout | [0.1, 0.2,0.3,0.4,0.5,0.6] |
|  | batch_size | [64,128, 256] |
| **Cox-Time** | |  |
|  | num_node | [64, 32], [64, 64], [64,128] |
|  | dropout | [0.1, 0.2,0.3,0.4,0.5,0.6] |
|  | batch_size | [64,128, 256} |
|  | num_duration | {10,15,30,50} |
| The table summarizes the hyper-parameter search ranges used for three machine learning survival models: Random Survival Forest (RSF), DeepSurv, and Cox-Time. For each model, the hyper-parameters and their corresponding candidate values used in the grid search or optimization procedure are listed. ML: Machine Learning; RSF: Random Survival Forest | | |
